# Copy Number Variation of Circulating Tumor DNA (ctDNA) Detected Using NIPT in Neoadjuvant Chemotherapy-Treated Ovarian Cancer Patients

**DOI:** 10.3389/fgene.2022.938985

**Published:** 2022-07-22

**Authors:** Mina Sharbatoghli, Fahimeh Fattahi, Hamidreza Aboulkheyr Es, Arvand Akbari, Setareh Akhavan, Marzieh Ebrahimi, Mohsen Asadi-Lari, Mehdi Totonchi, Zahra Madjd

**Affiliations:** ^1^ Oncopathology Research Center, Iran University of Medical Sciences (IUMS), Tehran, Iran; ^2^ Department of Embryology, Reproductive Biomedicine Research Center, Royan Institute for Reproductive Biomedicine, ACECR, Tehran, Iran; ^3^ School of Biomedical Engineering, University of Technology Sydney, Sydney, NSW, Australia; ^4^ Department of Genetics, Reproductive Biomedicine Research Center, Royan Institute for Reproductive Biomedicine, ACECR, Tehran, Iran; ^5^ Department of Gynecologic Oncology, Vali-Asr Hospital, Tehran University of Medical Sciences, Tehran, Iran; ^6^ Department of Stem Cells and Developmental Biology, Cell Science Research Center, Royan Institute for Stem Cell Biology and Technology, ACECR, Tehran, Iran; ^7^ Department of Epidemiology, School of Public Health, Iran University of Medical Sciences, Tehran, Iran; ^8^ Department of Molecular Medicine, Faculty of Advanced Technologies in Medicine, Iran University of Medical Sciences, Tehran, Iran

**Keywords:** NIPT, ctDNA, CNVs, ovarian cancer, copy number variations

## Abstract

Analysis of circulating tumor DNA (ctDNA) can be used to characterize and monitor cancers. Recently, non-invasive prenatal testing (NIPT) as a new next-generation sequencing (NGS)-based approach has been applied for detecting ctDNA. This study aimed to investigate the copy number variations (CNVs) utilizing the non-invasive prenatal testing in plasma ctDNA from ovarian cancer (OC) patients who were treated with neoadjuvant chemotherapy (NAC). The plasma samples of six patients, including stages II–IV, were collected during the pre- and post-NAC treatment that were divided into NAC-sensitive and NAC-resistant groups during the follow-up time. CNV analysis was performed using the NIPT via two methods “an open-source algorithm WISECONDORX and NextGENe software.” Results of these methods were compared in pre- and post-NAC of OC patients. Finally, bioinformatics tools were used for data mining from The Cancer Genome Atlas (TCGA) to investigate CNVs in OC patients. WISECONDORX analysis indicated fewer CNV changes on chromosomes before treatment in the NAC-sensitive rather than NAC-resistant patients. NextGENe data indicated that CNVs are not only observed in the coding genes but also in non-coding genes. CNVs in six genes were identified, including HSF1, TMEM249, MROH1, GSTT2B, ABR, and NOMO2, only in NAC-resistant patients. The comparison of these six genes in NAC-resistant patients with The Cancer Genome Atlas data illustrated that the total alteration frequency is amplification, and the highest incidence of the CNVs (≥35% based on TCGA data) is found in MROH1, TMEM249, and HSF1 genes on the chromosome (Chr) 8. Based on TCGA data, survival analysis showed a significant reduction in the overall survival among chemotherapy-resistant patients as well as a high expression level of these three genes compared to that of sensitive samples (all, *p* < 0.0001). The continued Chr8 study using WISECONDORX revealed CNV modifications in NAC-resistant patients prior to NAC therapy, but no CNV changes were observed in NAC-sensitive individuals. Our findings showed that low coverage whole-genome sequencing analysis used for NIPT could identify CNVs in ctDNA of OC patients before and after chemotherapy. These CNVs are different in NAC-sensitive and -resistant patients highlighting the potential application of this approach in cancer patient management.

## Introduction

Ovarian cancer (OC) is a tumor with the worst prognosis among female malignancies, and most cases are diagnosed in the advanced stage with peritoneal metastases (Bray et al., 2018). Neoadjuvant chemotherapy (NAC) is the gold standard treatment for OC patients with a high perioperative risk profile and/or a poor chance of effective debulking surgery (Elies et al., 2018), although a considerable percentage of patients demonstrate resistance to NAC treatment (Sato and Itamochi, 2014). Considering that the administration of proper chemotherapy regimens is dependent on imaging tests, cytology, and laparoscopic biopsy (Lee et al., 2018), inadequate tumor specimens are a flaw and difficulty in cytology or laparoscopic biopsy prior to the onset of NAC (Sharbatoghli et al., 2020). Biomarkers related to drug resistance and treatment, including prognostic and predictive molecules, play a key role in selecting appropriate treatment protocols and improving survival rates (Le Page et al., 2010). In OC, several studies have demonstrated changes in the serum levels of cancer antigen 125 (CA125) which may serve as a predictor of monitoring the response to NAC (Pelissier et al., 2014; Zeng et al., 2016), but its utility is often limited (Sharbatoghli et al., 2020). Serum tumor markers do not exhibit a significant increase in some histological types of OC (Sharbatoghli et al., 2020). Since gene amplification and deletion are common in cancer cells and contribute to cancer cell growth, angiogenesis, and drug resistance (Matsui et al., 2013; Rahimi et al., 2020), recently, copy number variants (CNVs) have been reported as potential biomarkers in cancer management (Pan et al., 2019; Yu et al., 2019; Jin et al., 2021). Moreover, comparing gene expression, CNV is considerably more stable and robust (Pan et al., 2019; Shao et al., 2019) and the gain or loss of gene copies often correlates with a corresponding increase or decrease in the amount of RNA and protein encoded by the gene (Hastings et al., 2009). It is critical to accurately measure copy number alterations in tumor samples in order to enable translational research and precision medicine. The majority of the CNV investigations were conducted on tumor tissue biopsy samples (Vives-Usano et al., 2021). A fraction of cell-free DNA (cfDNA) in cancer patients originates from tumor cells, known as circulating tumor DNA (ctDNA), which is obtained through liquid biopsy. Liquid biopsy as a semi-invasive diagnostic and prognostic tool has the advantage of being less invasive than tumor biopsy, and specimens can be frequently checked in real-time (Mathai et al., 2019). It seems that the molecular alterations identified in ctDNA might mirror molecular heterogeneity of tumor compared to those reflected by tumor biopsy (Sharbatoghli et al., 2020). Therefore, ctDNA analysis was applied to detect various types of genomic alterations, such as CNVs, mutation, and nucleosome positioning variation in cancers (Molparia et al., 2017; Huang et al., 2019; Noguchi et al., 2020).

In recent years, cancer detection by non-invasive prenatal testing (NIPT) as a new next-generation sequencing (NGS)-based approach is used to detect ctDNA (Cohen et al., 2016; Nakabayashi et al., 2018). The low coverage of whole-genome sequencing of cfDNA from maternal plasma is the basis for prenatal screening of common fetal autosomal aneuploidies and trisomy of chromosomes 21, 18, and 13 utilizing NIPT. One of the most often publicized benefits of the NIPT for chromosomal abnormality screening in pregnant women is its low false-positive rate (1–3%) (Filoche et al., 2017). Regarding fast advances in the NIPT, analysis of tumor CNV changes in ctDNA using NIPT was introduced as a potential cancer screening tool. In this context, Amant et al. (2015) discovered cancer in three pregnant women who had NIPT and indicated that CNVs may be used as a cancer screening tool. Furthermore, the implications of the whole-genome NIPT platform for cancer screening were shown in OC patients (Cohen et al., 2016). Furthermore, CNV analysis in cell-free DNA by low coverage whole-genome sequencing was used as a biomarker for the diagnosis of OC (Vanderstichele et al., 2017). For the first time, we applied the NIPT as a non-invasive test platform to compare the tumor-derived CNV in ctDNA measured pre- and post-NAC in plasma samples obtained from OC patients. This is proof of the concept that NIPT might be useful for predicting responsive and resistant patients to chemotherapy.

## Materials and Methods

### Collection of Samples and Blood Processing

This study was conducted as a prospective study with 10 plasma samples derived from 6 OC patients of the Cancer Institute of Imam Khomeini Hospital (Tehran, Iran) between December 2018 and October 2019. This study was performed with the approval of the Ethics Committee of Iran University of Medical Sciences (authorization no: IR.IUMS.REC 1397.32825). Each participating hospital’s ethical norms required that written informed permission has to be acquired. The FIGO (International Federation of Gynecology and Obstetrics) stage (Bhatla and Denny, 2018) was used to histologically confirm the patients’ diagnosis. Blood samples were obtained in pre- and post-NAC treatment from OC patients that received platinum-based chemotherapy as an NAC regimen, at the first-line treatment. A week before the first dose of chemotherapy, baseline blood samples were collected, and post-NAC samples were taken after the first course (six cycles) of chemotherapy. The patients with complete response were defined as NAC-sensitive, while those with stable disease and progressive disease were defined as NAC-resistant (Noguchi et al., 2020). A total volume of 10 ml of whole blood was collected in K_2_EDTA-coated tubes (REF: CDLP 029, C.D. RICH^®^, Romania) from each patient. Then, the blood was centrifuged at 1,600×g for 15 min at 25°C, and the plasma fraction was collected and centrifuged for a second time at 2,500×g for 10 min at 25°C. After the second spin, the plasma was transferred into barcoded tubes and immediately stored at ≤-70°C. The cell-free DNA was extracted from 3 ml of patient plasma using the QIAamp Circulating Nucleic Acid Kit (Qiagen, Gaithersburg, MD, United States) (Diefenbach et al., 2018).

### Library Preparation, Sequencing, and Data Analysis

DNA libraries were prepared from 2 ng of cell-free DNA extracted from 3 ml of plasma using the VeriSeq NIPT Solution v2 according to the manufacturer’s instructions for 75 bp single-end high via sequencing. All libraries were normalized to 1.6 nM, multiplexed, and sequenced on HiSeq: 4000 with 27 sequencing cycles of the cell-free DNA insert and an additional eight sequencing cycles for the index barcodes. Each research sample was sequenced alongside 12 clinical samples, with 36-cycle single-end sequencing on an Illumina NextSeq550. The read depth was low coverage at 0.2× to 0.3× based on the amount of sequencing data. The open-source algorithm WISECONDORX (WIthin-SamplE COpy Number Aberration DetectOR X) and NextGENe (Next GENeration sequencing software for biologists) were used for data analysis (Faircloth and Glenn, 2012).

### Copy Number Variation Call Using WISECONDORX and NextGENe

WISECONDORX was used to identify whole chromosome (Chr) and subchromosomal abnormalities that the standard NIPT pipeline failed to detect (Raman et al., 2019). Segmental alterations of less than 0.05 Mb were prespecified as abnormal (“positive cancer screen”). FASTQC (Andrews, 2010) was used to do quality control on the raw single-end sequencing data. The BBduk tool from the BBmap toolbox (Bushnell, 2014) was used to trim and adjust the fastq files as needed.

Reads were mapped to the human reference genome (hg38) using bwa samse algorithm (Li and Durbin, 2009). The resulting sam files were subjected to corrections and converted to bam via samtools (Li et al., 2009). CNVs were called by WISECONDORX run on default settings suggested by the developer (Raman et al., 2018). NextGene software version 2.4.1 (SoftGenetics, LLC) was used for CNV analysis according to the Kerkhof et al. (2017) method.

After obtaining WISECONDORX and NextGENe data, an investigation of these CNV results in pre-treatment was performed on all patients. Moreover, a comparison of these data for patients within the NAC-sensitive and NAC-resistant groups were performed separately to obtain common alterations in chromosomes and gene levels for each group. Then, the data of WISECONDORX and NextGENe software from NAC-resistant were compared to NAC-sensitive patients in order to receive alteration genes detected in NAC resistance. Similarly, post-treatment results were compared in patients with OC in order to detect CNVs.

### Data Mining for Genes Detected in NAC-Resistant Patients

To obtain a more comprehensive and deep understanding of the biological process and molecular function of genes detected in NAC resistance, Gene Ontology (GO) term enrichment analysis was performed (Ashburner et al., 2000). Furthermore, KEGG (Kanehisa and Goto, 2000), Reactome (Sidiropoulos et al., 2017), and WikiPathways (Slenter et al., 2018) were investigated to examine the biological pathways in which these genes are involved. GO enrichment and pathway analysis of the genes detected in NAC-resistant patients was revealed using the ClueGO plug-in using Cytoscape (Bindea et al., 2009). Furthermore, genes detected in NAC resistance were investigated in the cBio Cancer Genomics Portal (cBioPortal) database in order to further evaluate the alterations of these genes in OC tissue samples. cBioPortal is an open-access database providing visualization and analyzing tools for multidimensional cancer genomics data, such as The Cancer Genome Atlas (TCGA) (Cerami et al., 2012). The genes detected in NAC resistance with more copy number alteration frequency on CBioPortal were selected to evaluate the mRNA expression levels on Gene Expression Profiling Interactive Analysis (GEPIA2) for OC tissue data compared to normal tissues (Tang et al., 2019). GEPIA2 is an online database, including RNA sequence expression data based on tumor and normal samples from TCGA and the GTEx (Tang et al., 2019). Gene Set Cancer Analysis (GSCALite) was applied to investigate the correlation between mRNA expression and CNV in OC patients. Spearman correlation coefficients were reported using GSCALite software, a user-friendly web server for dynamic analysis and visualization of gene sets in cancer which will be of broad utility to cancer researchers (Liu et al., 2018) from TCGA (Liu et al., 2018). The Cancer Virtual Cohort Discovery Analyses Platform (CVCDAP) portal was also utilized to compare the overall survival (OS) analyses between chemo-resistant and chemo-sensitive patients based on mRNA expression levels of these genes (Guan et al., 2020). CVCDAP is a web-based platform to deliver an interactive and customizable toolbox off the shelf for cohort-level analysis of TCGA and CPTAC public datasets (Guan et al., 2020). Finally, a volcano plot was applied to evaluate the protein expression in OC chemotherapy-resistant patients compared to that in chemotherapy-sensitive patients by TCGAbiolinks, an R/Bioconductor package for integrative analysis of TCGA data (Colaprico et al., 2016).

## Results

### Patient Characteristics

The clinicopathological characteristics and response to NAC of the six OC patients are summarized in Table 1. The mean age of the patients was 54.3 years (38–78 years), and they included stages II, III, and IV high-grade serous ovarian carcinoma (HGSOC). Two of the six patients died undergoing chemotherapy. As a result, these two patients were unable to provide post-NAC plasma samples. Two patients were NAC-sensitive, while two cases were NAC-resistant and did not respond appropriately to chemotherapy treatment.

**TABLE 1:** Clinicopathological characteristics of six ovarian cancer patients that were treated with neoadjuvant chemotherapy.

Case no.	Age (Year)	FIGO stage	Right (R) and left (L) ovary	Histological diagnosis	Treatment	Clinical response to therapy
1	38	III	R	HGSOC	Carboplatin + paclitaxel	Progressive disease
2	78	IV	R	HGSOC	Carboplatin + paclitaxel	Responsive
3	45	II	R	HGSOC	Carboplatin + paclitaxel	Responsive
4	50	III	R and L	HGSOC	Carboplatin + paclitaxel	Progressive disease
5	54	III	R and L	HGSOC	Carboplatin + paclitaxel	Progressive disease (death)
6	68	IV	R	HGSOC	Carboplatin + paclitaxel	Progressive disease (death)

### Investigation of CNV From ctDNA in NAC-Resistant and NAC-Sensitive Patients With OC

**FIGURE 1:**
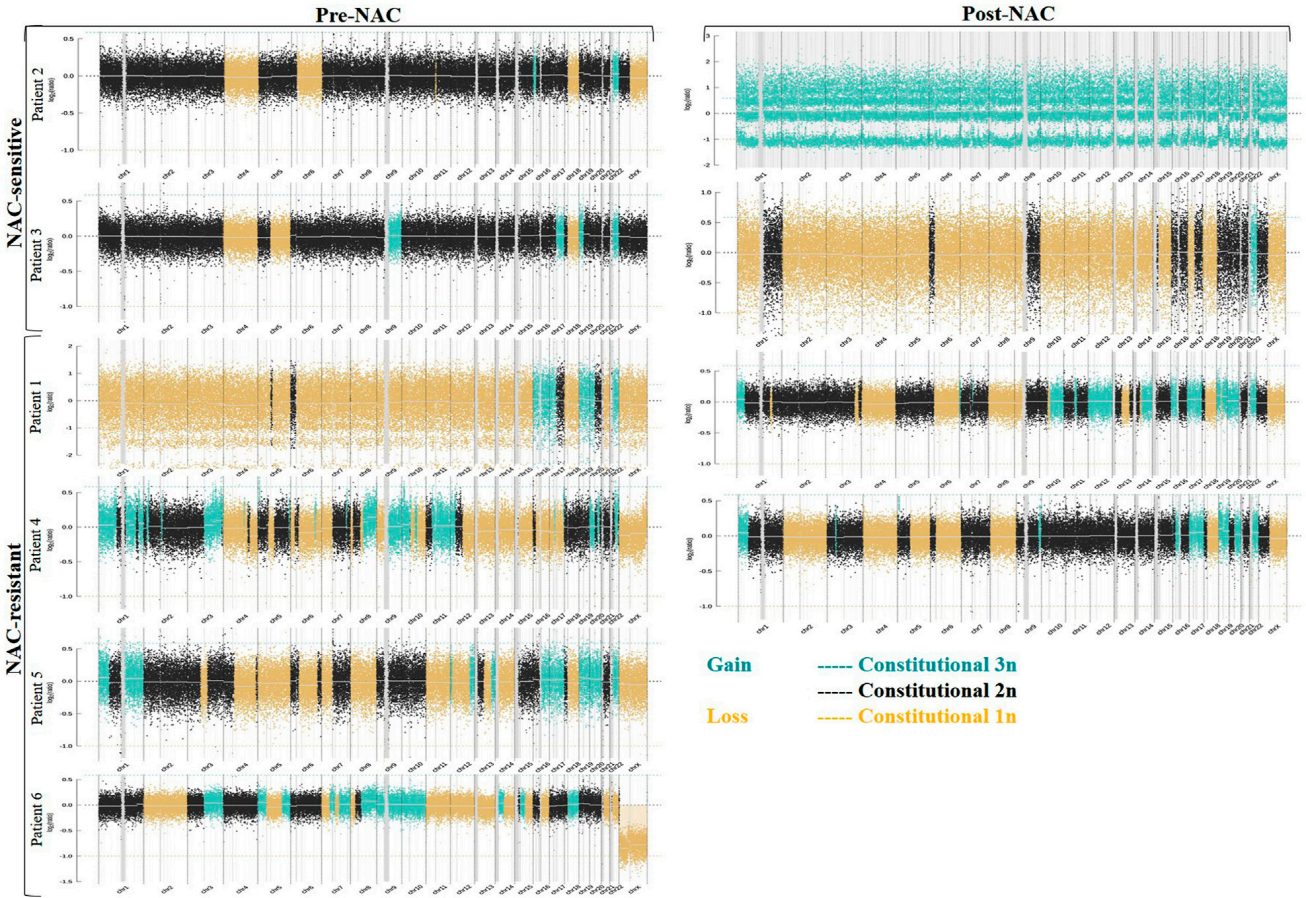
Copy number variations (CNVs) in ctDNA from six ovarian cancer (OC) patients that were treated with neoadjuvant chemotherapy (NAC). Data CNVs for all chromosomes based on WISECONDORX analysis were detected from NAC-sensitive, NAC-resistant, and dead patient groups. The copy numbers were segmented in blue (gain) and yellow (loss) lines.

We detected 6/6 HGSOC cases including early-stage (II) to late-stage (IV)—using the WISECONDORX (Table 2) and NextGENe analysis (Supplementary Tables S1–S3). WISECONDORX results indicated that most patients (at least 5 cases) have abnormality (gain and/or loss) on chromosomes, 4, 9, 18, and 22 before NAC treatment (Table 2). NextGENe data illustrated CNVs not only in coding genes but also alterations in duplication and deletion in non-coding genes (Supplementary Tables S1–S3). Chr changes detected by WISECONDORX software indicated that NAC-sensitive patients have fewer chromosomes’ CNV changes before treatment rather than NAC-resistant patients as shown in Figure 1; Table 2.

**TABLE 2:** Copy number variations (CNVs) in six ovarian cancer cases which were reported based on gains and losses of chromosomes by the WISECONDORX analysis.

Patient 1		Patient 2		Patient 3		Patient 4		Patient 5		Patient 6	
Pre-NAC	Post-NAC	Pre-NAC	Post-NAC	Pre-NAC	Post-NAC	Pre-NAC	Post-NAC	Pre-NAC	Post-NAC	Pre-NAC	Post-NAC
Chr1 loss	Chr1 gain and loss	Chr4 loss	Chr1 gain	Chr4 loss	Chr1 loss	Chr1 gain	Chr1 gain	Chr1 gain	No data	Chr2 loss	No data
Chr2 loss	Chr3 loss	Chr6 loss	Chr2 gain	Chr5 loss	Chr2 loss	Chr2 gain	Chr2 loss	Chr3 loss		Chr3 gain	
Chr3 loss	Chr4 loss	Chr11 loss	Chr3 gain	Chr9 gain	Chr3 loss	Chr3 gain	Chr3 gain	Chr4 loss		Chr5 gain and loss	
Chr4 loss	Chr6 loss	Chr16 gain	Chr4 gain	Chr17 gain	Chr4 loss	Chr4 gain and loss	Chr4 loss	Chr5 loss		Chr7 gain and loss	
Chr5 loss	Chr7 gain	Chr18 loss	Chr5 gain	Chr18 loss	Chr5 loss	Chr5 gain and loss	Chr5 gain and loss	Chr6 loss		Chr8 gain and loss	
Chr6 loss	Chr8 loss	Chr22 gain	Chr6 gain	Chr19 gain	Chr6 loss	Chr6 gain and loss	Chr6 loss	Chr7 loss		**Chr9 gain**	
Chr7 loss	Chr9 loss	ChrX loss	Chr7 gain	Chr22 gain	Chr7 loss	Chr7 gain and loss	Chr8 loss	Chr8 loss		Chr10 gain	
Chr8 loss	Chr10 gain and loss		Chr8 gain		Chr8 loss	Chr8 gain and loss	Chr9 gain	Chr9 loss		Chr11 loss	
Chr9 loss	Chr11 gain		Chr9 gain		Chr9 loss	Chr9 gain and loss	Chr16 gain	Chr11 loss		Chr12 loss	
Chr10 loss	Chr12 gain		Chr10 gain		Chr10 loss	Chr10 gain and loss	Chr17 gain	Chr12 gain and loss		Chr13 loss	
Chr11 loss	Chr13 loss		Chr11 gain		Chr11 loss	Chr11 gain	Chr18 loss	Chr13 gain and loss		Chr14 gain and loss	
Chr12 loss	Chr14 gain and loss		Chr12 gain		Chr12 loss	Chr12 gain and loss	Chr19 gain	Chr14 loss		Chr15 gain and loss	
Chr13 loss	Chr16 gain		Chr13 gain		Chr13 loss	Chr13 loss	Chr20 gain	Chr16 gain		Chr16 loss	
Chr14 loss	Chr17 gain		Chr14 gain		Chr14 loss	Chr14 loss	Chr22 gain and loss	Chr17 gain		Chr18 gain	
Chr15 gain and loss	Chr18 loss		Chr15 gain		Chr15 loss	Chr15 loss	ChrX loss	Chr18 loss		Chr21 loss	
Chr16 gain	Chr19 gain and loss		Chr16 gain		Chr17 loss	Chr16 loss		Chr19 gain		Chr22 loss	
Chr17 gain	Chr20 gain		Chr17 gain		Chr18 loss	Chr17 gain and loss		Chr20 gain		ChrX gain and loss	
Chr18 loss	Chr22 gain		Chr18 gain		Chr22 gain	Chr20 gain		Chr22 gain			
Chr19 gain	ChrX loss		Chr19 gain		ChrX loss	Chr22 gain		ChrX loss			
Chr20 gain			Chr20 gain			ChrX loss					
Chr21 loss			Chr21 gain								
Chr22 gain			Chr22 gain								
ChrX loss			ChrX gain								

**FIGURE 2:**
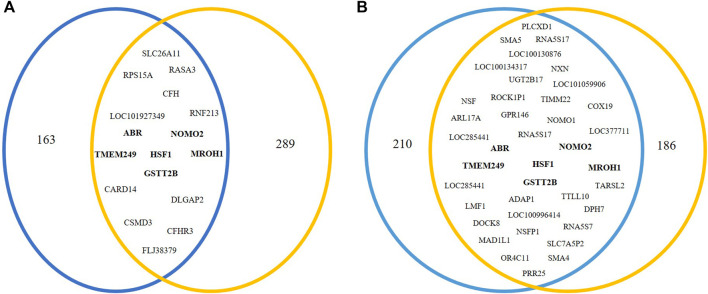
Venn diagram analysis to investigate genes related to NAC resistance. In NAC-resistant patients, Venn diagram indicated **(A)** 17 common genes in pre-NAC-treated patients and **(B)** 38 common genes in post-NAC-treated patients that were obtained via NextGENe analysis.

Using the results of NextGENe software, common genes with copy number changes were assessed in pre-NAC and post-NAC treatment by the Vinny plot. We detected 17 common genes with CNV in pre-NAC patients from the NAC-resistant group (Figure 2A; Supplementary Table S4). Among these genes, six common genes, including NOMO2, ABR, GSTT2B, HSF1, TMEM249, and MROH1, exclusively have shown CNV in the NAC-resistant group, while in NAC-sensitive patients, none of these genes were altered before NAC treatment. Furthermore, as shown in Figure 2B; Supplementary Table S5, 38 common genes with CNV were discovered in NAC-resistant patients’ post-NAC data. Furthermore, we discovered 14 genes, including LOC285441, LOC100996414, TTLL10, NXN, SMA4, SMA5, NOMO1, TIMM22, NOMO2, HSF1, ABR, TMEM249, GSTT2B, and MROH1, that exclusively revealed CNVs in NAC-resistant patients following chemotherapy. We indicate the CNVs for the six genes (NOMO2, ABR, GSTT2B, HSF1, TMEM249, and MROH1) that were frequent in the NAC-resistant group pre- and post-treatment as genes associated and found in NAC-resistant patients (See Table 3).

**TABLE 3:** Common genes in pre- and post-neoadjuvant chemotherapy (NAC) as well as its copy number variation (CNV) status (in pre-and post-NAC) for NAC-resistant patients that were detected by NextGENe software.

Common gene	Patient 1 CNV status		Patient 4 CNV status	
	Pre-NAC	Post-NAC	Pre-NAC	Post-NAC
NOMO2 (Chr16)	Dup	Dup	Dup	Dup
ABR (Chr17)	Dup	Dup	Dup	Dup
GSTT2B (Chr22)	Dup	Dup	Dup	Dup
MROH1 (Chr8)	Dup	Normal	Dup	Dup
HSF1 (Chr8)	Dup	Normal	Dup	Dup
TMEM249 (Chr8)	Dup	Normal	Dup	Dup

Dup, Duplication

### Data Mining Approaches for Genes Detected in NAC-Resistant Patients

**FIGURE 3:**
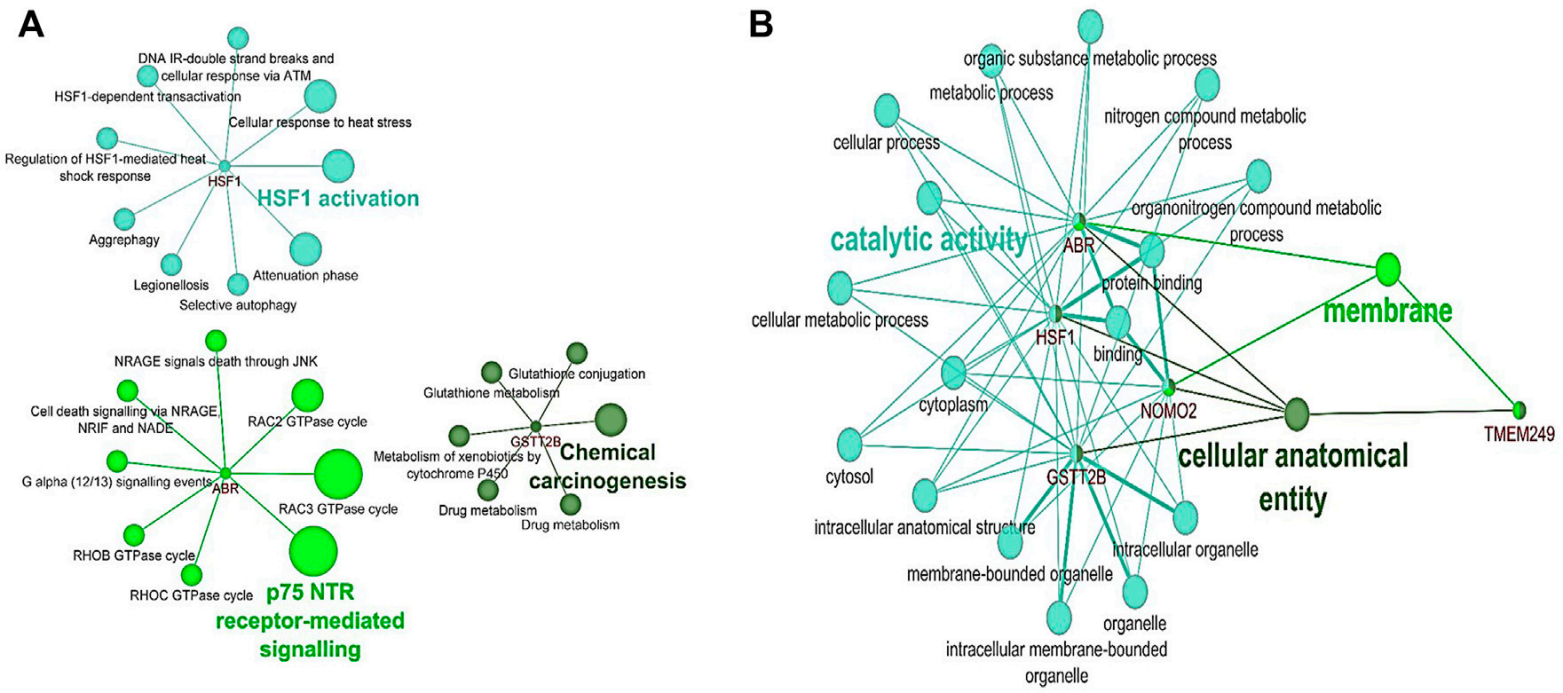
Pathway and Gene Ontology analysis for six genes detected in NAC-resistant patients using the ClueGO plugin in Cytoscape. **(A)** Pathway analysis based on KEGG, Reactome, and WikiPathways. **(B)** Common results of GO analysis for HSF1, ABR, and GSTT2B according to biological processes and molecular function.

The pathway enrichment analysis for six common genes detected in NAC-resistant patients indicated that GSTT2B was involved in chemical carcinogenesis and drug metabolism pathways, while ABR and HSF1 were enriched in p75 NTR receptor-mediating signaling and cellular response to heat stress/shock response pathways, respectively (Figure 3A). Gene Ontology analysis illustrated that ABR and HSF1 contributed to apoptosis and programmed cell death (GO: 0012501 and 0006915, respectively) (Supplementary Table S6). Moreover, ABR, GSTT2B, and HSF1 have a role in catalytic activity and metabolic processes (Figure 3B).

**FIGURE 4:**
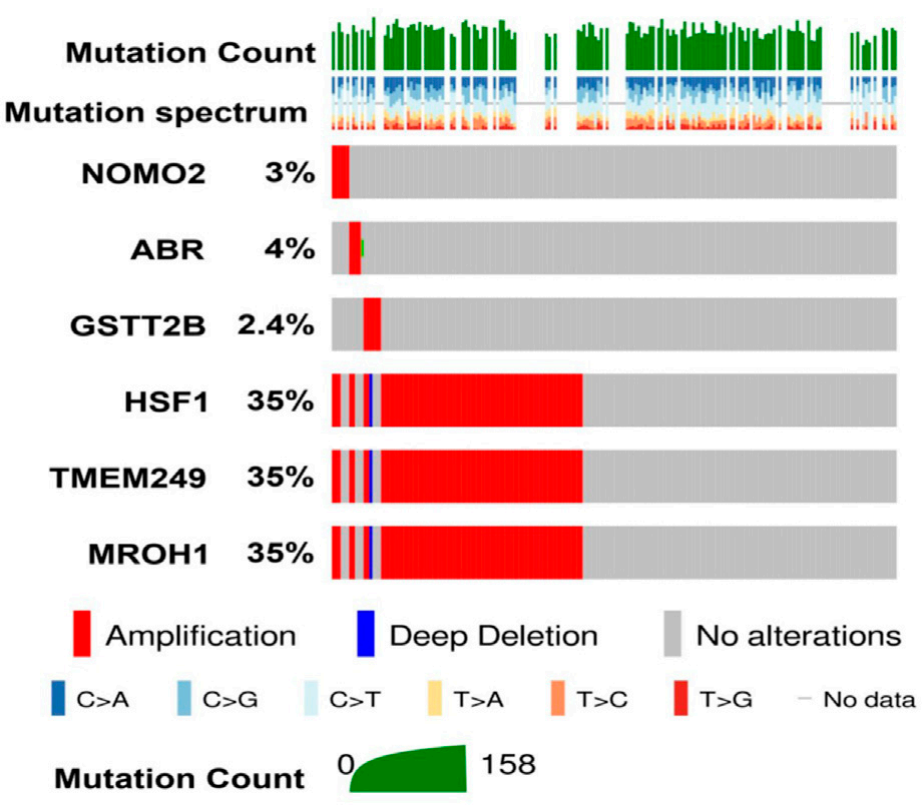
Investigation of DNA alterations for six genes detected in NAC resistance in TCGA-ovarian cancer patients on cBioPortal. Oncoplot illustrates the amplification status of six different genes identified in resistant patients across 585 ovarian carcinoma patients from TCGA. Among them, high amplification of HSF1, TMEM249, and MROH1 was observed compared to other identified genes.

Comparing our CNV results of six common genes detected in NAC-resistant patients with TCGA data through the cBioPortal illustrated that the total alteration frequency is amplification, similar to TCGA data from 585 OC patients (Figure 4). Moreover, the most alteration frequency of CNVs (≥35%) was observed in HSF1, TMEM249, and MROH1 genes from OC patients (Figure 4).

**FIGURE 5:**
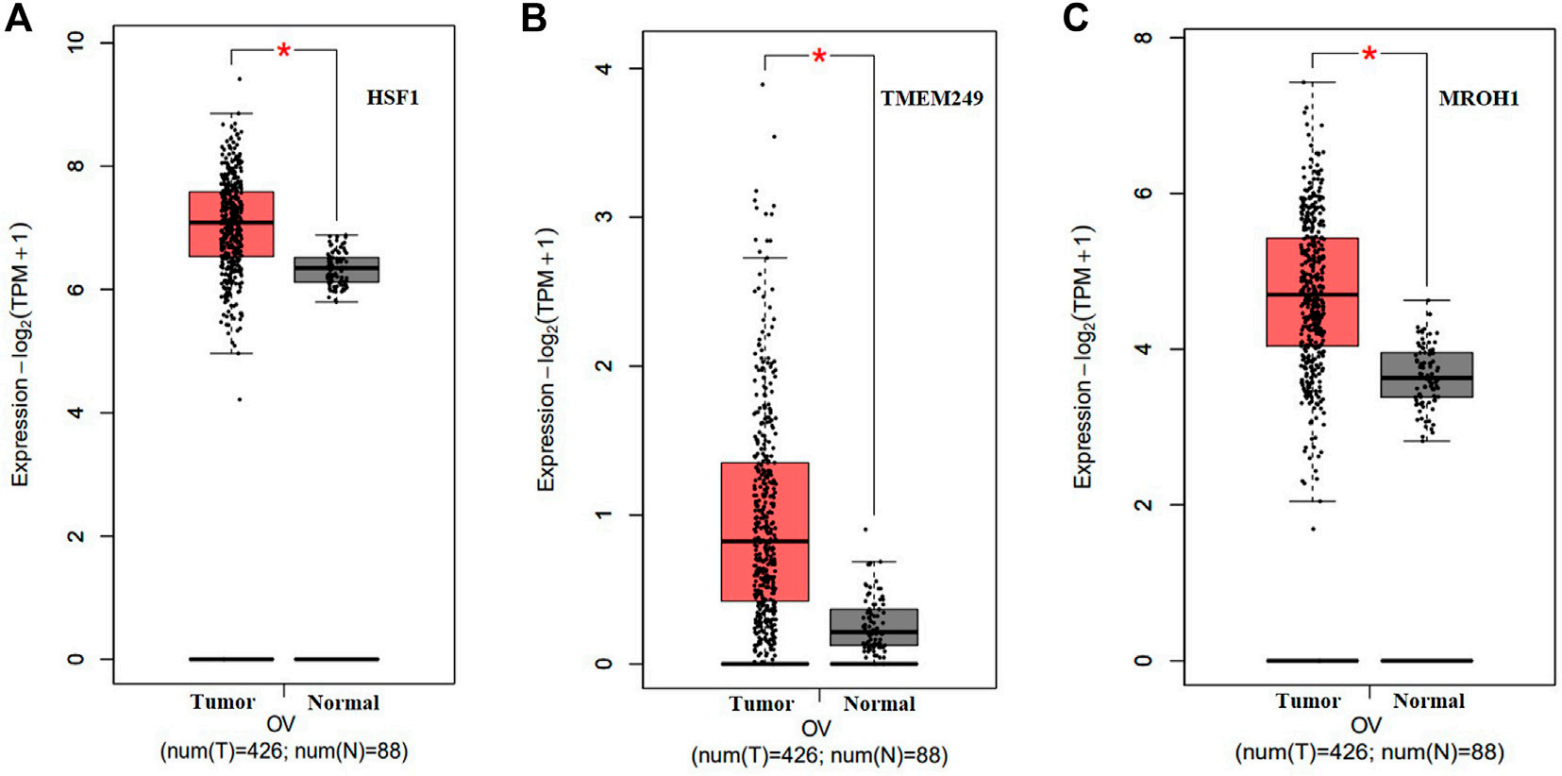
mRNA levels of three genes related to NAC resistance in serous ovarian carcinoma (SOC) on Gene Expression Profiling Interactive Analysis2 (GEPIA2). Upregulated expression of **(A)** HSF1, **(B)** TMEM249, and **(C)** MROH1 in mRNA levels significantly for SOC compared with that for normal tissue by GEPIA2 was observed (*: *p < 0.05*).

**FIGURE 6:**
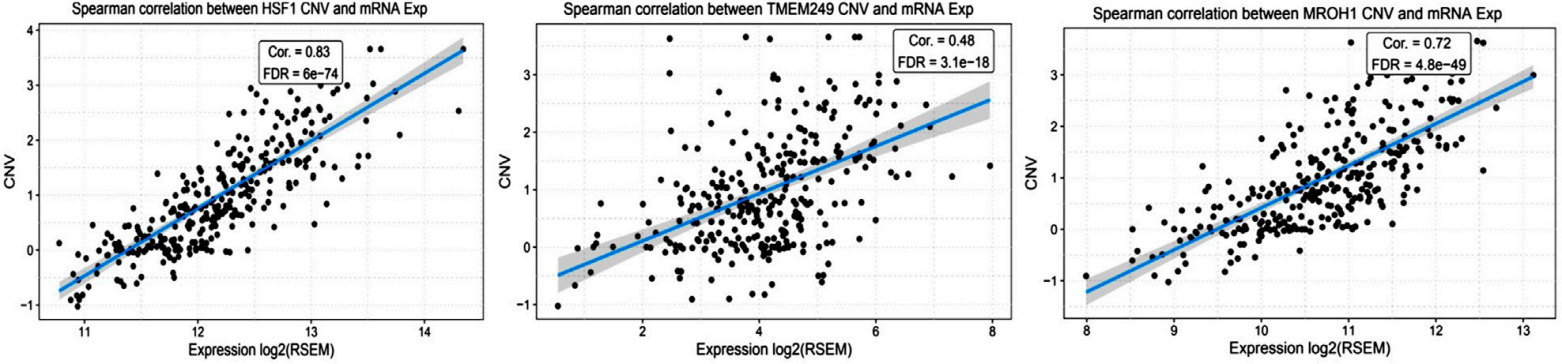
Correlation study between mRNA levels and copy number variation of three genes related to NAC resistance through cBioPortal in TCGA-ovarian cancer patients. The correlation coefficient analysis shows the positive association between mRNA expression and copy number variation of HSF1, TMEM249, and MROH1 across 585 ovarian cancer patients from TCGA.

**FIGURE 7:**
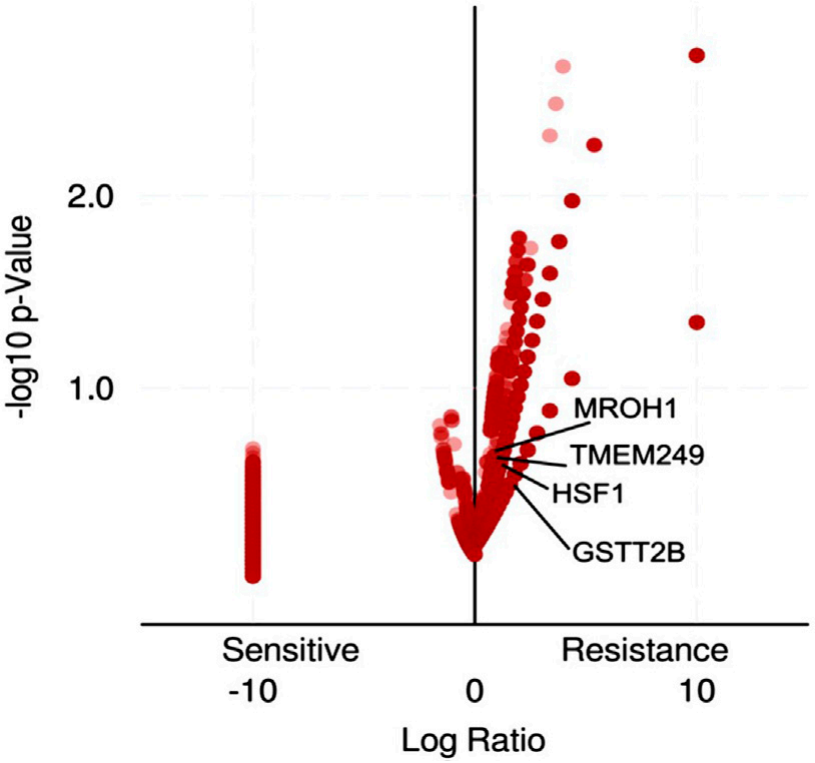
Volcano plot enrichment analysis for gene-related protein expression in ovarian cancer patients who were treated with chemotherapy from TCGA using TCGAbiolinks. The expression of HSF1, TMEM249, and MROH1 proteins significantly increased in resistant patients (*n* = 90) versus sensitive patients to chemotherapy (*n* = 194).

Loci of HSF1, TMEM249, and MROH1 genes are arranged on Chr8. CNV alterations in Chr8 in WISECONDORX data were solely found in NAC-resistant patients when compared to NAC-sensitive patients before treatment (Table.2). Investigation of these three genes at the mRNA level on GEPIA2 showed significantly high expression of HSF1, TMEM249, and MROH1 in ovarian serous carcinoma (OSC) compared with normal tissues (all, *p < 0.05*) (Figure 5A–C). Moreover, the Spearman correlation coefficient shows the positive association between CNVs and mRNA expression of HSF1 (Cor = 0.83, FDR = 6e-74), MROH1 (Cor = 0.72, FDR = 4.8e-49), and TMEM249 (Cor = 0.48, FDR = 3.11e-18) across 585 OC patients from TCGA (Figure 6). As shown in Figure 7, the volcano plot indicates the enrichment of TMEM249, MROH1, and HSF1 protein expression in resistant patients to chemotherapy from TCGA. In other words, the expression of these three proteins significantly increased in resistant patients (*n* = 90) versus sensitive patients to chemotherapy (*n* = 194) (all, *p < 0.05*).

**FIGURE 8:**
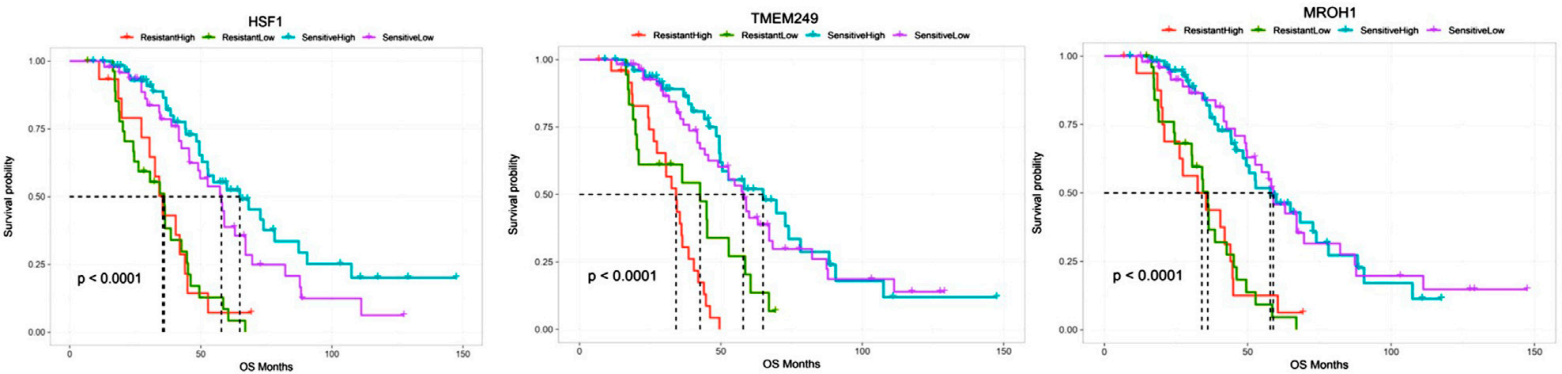
Comparing the overall survival between chemo-resistant and -sensitive expression of HSF1, TMEM249, and MROH1 using the CVCDAP portal for ovarian cancer patients. The plot shows a significant reduction in the overall survival across patients resistant to chemotherapy and expressed a high level of HSF1, TMEM249, and MROH1 genes in comparison with sensitive samples. Moreover, the plot indicates that high expression of MROH1 and TMEM249 in chemo-sensitive samples can reduce OS among these patients.

Survival analyses showed a significant reduction in OS across patients resistant to chemotherapy and a high expressed level of HSF1, TMEM249, and MROH1 compared to sensitive samples (all, *p < 0.0001*). Moreover, these data illustrate that high expression of MROH1 and TMEM249 significantly in resistant and sensitive samples can reduce OS among these patients (all, *p < 0.0001*) (Figure 8).

## Discussion

ctDNA from liquid biopsy has recently been shown to be a useful diagnostic tool in a variety of cancer patients (Saha et al., 2022). Indeed, it has been shown that ctDNA is correlated to tumor burden and the risk of recurrence (Fleischhacker and Schmidt, 2007). ctDNA as part of total body DNA from cancer cells is superior to other plasma biomarkers such as RNA and protein. It is more stable than RNA (Panawala, 2017) with more sensitivity and clinical correlations. Despite the fact that plasma protein biomarkers are commonly used in clinical management for different cancers, such as AFP, CEA, PSA, and CA15-3 (Mazzucchelli et al., 2000; He et al., 2013), some cancer patients are not positive for these biomarkers. Furthermore, they are found with lower concentrations in the serum of individuals free of cancer (Cheng et al., 2016), while the studies show that ctDNA can more accurately reflect the real-time tumor burden in patients receiving therapy (Bettegowda et al., 2014).

The current study is the first report to indicate the utility of analyzing CNVs in ctDNA using massively parallel sequencing (such as NIPT), before and after NAC treatment in OC patients. The previous study by Fleischhacker and Schmidt, (2007) has shown that ctDNA is low in the early stages of the tumor and difficult to detect. The method in this study can detect copy number alterations of ctDNA in various stages of HGSOC patients, including stages II–IV, through low coverage plasma DNA sequencing and analysis for chromosomal CNVs ≥0.5 Mb. It has previously been demonstrated that tumor DNA from cancer cells has been detected in plasma using NIPT (Amant et al., 2015; Bianchi et al., 2015; Cohen et al., 2016). We also investigated the response to chemotherapy and depicted the CNV changes in NAC-sensitive and NAC-resistant patients. CNV burden in all chromosomes of NAC-sensitive patients was fewer than that of NAC-resistant patients before NAC treatment. Since the CNVs are considered a key factor in the genetic variation of tumors (Hu et al., 2021), it seems that less CNV burden indicated better response in treating patients (Walker et al., 2017). CNVs were also discovered in a variety of genes following chemotherapy, notably in genes that control drug absorption into cells and drug metabolism (Willyard, 2015). As a result, we compared produced CNVs in NAC-sensitive and NAC-resistant patient groups with OC following NAC therapy. After the first course of chemotherapy, the CNV load increased in both NAC-sensitive and NAC-resistant individuals, according to our findings. This phenomenon could be in terms of the death of tumor cells (DNA damage) (Woods and Turchi, 2013) or resistance to therapy (Alfarouk et al., 2015).

The previous studies have suggested the role of CNVs in genes related to drug resistance (Alfarouk et al., 2015; Willyard, 2015; Costa et al., 2017; Qidwai, 2020). Our findings appear to well indicate that some of the CNVs detected by the NIPT are different in NAC-resistant groups compared to NAC-sensitive groups of OC patients. We found some common genes, including NOMO2, ABR, GSTT2B, HSF1, TMEM249, and MROH1, in the pre- and post-treatment for the NAC-resistant group which was no longer detected in NAC-sensitive patients before and after therapy. These findings suggest that CNVs discovered by the NIPT may contribute to treatment resistance. To confirm the presence of CNV in all patients’ plasma, we matched the findings of genes with CNVs discovered in their plasma to the tissue samples in the TCGA data (Bell et al., 2011) owing to the unavailability of tissue specimens from these patients. It is better to investigate the plasma sequencing data with paired tumor DNA of tissue samples, but the lack of tissue or insufficient tumor tissue sampling of patients is a limitation for this type of study (Cohen et al., 2016). The cBioPortal findings indicated the existence of alteration and amplification in all six genes among TCGA patients so that some of these CNVs have a high frequency in OC tissue samples. Moreover, HSF1, TMEM249, and MROH1 are located on Chr8 which is highlighted in our study for OC patients. The CNVs’ investigation via WISECONDOREX shows Chr8 underwent CNV changes in NAC-resistant patients before NAC treatment, while in NAC-sensitive patients no CNV changes were found on Chr8 before chemotherapy. Amplification of Chr8 genes has also been identified as a recurrent genomic event in lung cancer (Baykara et al., 2015) and malignant peripheral nerve sheath tumor (MPNST) (Dehner et al., 2021). This finding indicates the power role and relationship among CNV alterations on Chr8 and NAC-resistant in OC patients. Other studies showed that approximately 80 genes on Chr8 are involved in cancer biology (Tabarés-Seisdedos and Rubenstein, 2009). We investigated the levels of RNA and protein expression from HSF1, TMEM249, and MROH1, which are related to NAC-resistant patients on TCGA data. DNA copy number variation is an important factor in the expression of genes (Gamazon and Stranger, 2015) and also an important influential factor for the expression of both protein-coding and non-coding genes (Liang et al., 2016). As expected, positive correlations between the level of mRNA and CNVs alterations were observed for HSF1, TMEM249, and MROH1. In this regard, survival analysis confirmed the influence expression of these three genes in the survival rate, so that resistant patients with higher mRNA expression of these genes had a reduced OS. Furthermore, in TCGA-chemotherapy-resistant OC patients, the protein expression of these genes was higher than that in sensitive individuals. So far, dysregulation and different roles of these genes in various cancers have been evaluated. Studies indicated that HSF1 has been implicated in tumorigenesis by its participation in cellular stress response pathways and its effect on regulatory pathways such as p53, mTOR, and insulin signaling (Vihervaara and Sistonen, 2014; Vydra et al., 2014; Powell et al., 2016; Barna et al., 2018). In line with our *in silico* analysis study, high levels of HSF1 have been identified in different types of cancers (Chen et al., 2021). Overexpression of HSF1 in tumor tissues is correlated with a worse prognosis in cancer patients (Kourtis et al., 2015). Regarding the correlation between a high level of HSF1 and the deterioration of disease in the initiation, promotion, and progression of cancer (Wang et al., 2020), HSF1 could act as a potential therapeutic target (Wang et al., 2020). OC studies reported that HSF1 induces epithelial–mesenchymal transition (EMT) in the *in vitro* models (Powell et al., 2016) and targeting HSF1 leads to an antitumor effect (Chen et al., 2017). Zhang et al. (2017) identified some parts of human Chr8, a location hotspot, mediated by the master regulator HSF1 in different cancers. Furthermore, they interestingly indicated MROH1 and TMEM249 immediately flanking the upstream and downstream regions of HSF1. As predicted, our CNV data for Chr8 genes, such as HSF1, MROH1, and TMEM249, were duplicated in individuals who were resistant to NAC. Considering HSF1’s critical involvement in cancer development, the lack of HSF1 in NAC-sensitive patients compared to NAC-resistant patients suggests a function for the Chr8 and HSF1 genes in NAC resistance. The activity of the GSTT2 enzyme is important for the protection of cells against toxic products of oxygen and lipid peroxidation (Tan and Board, 1996), which represents a major source of endogenous DNA damage in humans that contributes significantly to cancer and other genetic diseases (Marnett, 2002). Duplicated-CNV and also mRNA expression of GSTT2B in NAC-resistant patients by bioinformatics analysis are consistent with the previous cancer studies (Pool-Zobel et al., 2005; Doherty et al., 2014). Research on colon cancer cells indicated upregulated GSTT2 upon incubation with butyrate that is involved in defense against oxidative stress (Pool-Zobel et al., 2005). Furthermore, Doherty et al. (2014) observed that the expression of GSTT2 was increased in drug-resistant cervical cell models with cisplatin treatment.

Deletion of ABR indicated a tumor-suppressive role in several solid tumors, such as medulloblastoma (McDonald et al., 1994), astrocytomas (Willert et al., 1995), and breast cancer (Liscia et al., 1999). In acute myeloid leukemia, the ABR gene was detected as a prognostic factor in which the blockage of ABR prevents myeloid differentiation (Namasu et al., 2017). In our study, the ABR gene was duplicated in NAC-resistant patients before and after treatment. These data suggested more investigation into the role and function of the ABR gene that might influence resistance to chemotherapy for OC patients.

The NOMO2 gene is known as a diagnostic biomarker in radioresistance in human H460 lung cancer stem-like cells (Yun et al., 2016). We found NOMO2 duplication in NAC-resistant patients by NIPT. These data are consistent with the previous result in metastatic breast cancer by NGS that detected mutation or amplification in cfDNA samples (Page et al., 2017). CNVs were not only found in coding genes but also detected in non-coding genes, such as microRNA and long non-coding genes, although the previous study in bladder cancer indicated CNV alteration in long non-coding RNAs, which can be used as prognostic biomarkers for bladder cancer (Zhong et al., 2021). Furthermore, copy number changes in non-coding RNAs have been identified as prospective therapeutic targets and prognostic markers in lung squamous cell carcinoma (LUSC) (Ning et al., 2021). We are aware that our research has limitations to describe the biological behavior of these CNVs and their relationships with NAC resistance in OC cells. Although the number of records was adequate to establish a conclusion for the trend in data, however, in terms of seeking generalizability, larger sample sizes are strongly suggested, which could be covered via larger and multicenter investigations.

## Conclusion

This study’s findings highlighted low-coverage whole-genome sequencing analysis to investigate CNV changes in ctDNA. It seems that detected CNVs through the NIPT in ctDNA could be potential markers of clinical response to NAC treatment. Our results gave a clue that some alterations in the copy number of genes at the DNA level may relate to being the response to NAC treatment in OC patients, although further studies are warranted to understand the role of these CNVs in NAC-resistant patients. Using a platform for prenatal testing in diagnosis or monitoring therapeutic response for other cancer types as a novel way may hold promise that it should be examined.

## Data Availability

The data presented in the study are deposited in the European Nucleotide Archive (ENA) at EMBL-EBI under accession number PRJEB53061. (https://www.ebi.ac.uk/ena/browser/view/PRJEB53061).
